# Association between Gender, Process of Care Measures, and Outcomes in ACS in India: Results from the Detection and Management of Coronary Heart Disease (DEMAT) Registry

**DOI:** 10.1371/journal.pone.0062061

**Published:** 2013-04-24

**Authors:** Neha J. Pagidipati, Mark D. Huffman, Panniyammakal Jeemon, Rajeev Gupta, Prakash Negi, Thannikot M. Jaison, Satyavan Sharma, Nakul Sinha, Padinhare Mohanan, B. G. Muralidhara, Sasidharan Bijulal, Sivasubramonian Sivasankaran, Vijay K. Puri, Jacob Jose, K. Srinath Reddy, Dorairaj Prabhakaran

**Affiliations:** 1 Centre for Chronic Disease Control, New Delhi, India; 2 Division of Women’s Health, Brigham and Women’s Hospital, Boston, United States of America; 3 Department of Preventive Medicine, Northwestern University, Chicago, United States of America; 4 Institute of Cardiovascular and Medical Sciences, University of Glasgow, Glasgow, United Kingdom; 5 Public Health Foundation of India, New Delhi, India; 6 Department of Cardiology, Fortis Escorts Hospital, Jaipur, India; 7 Department of Cardiology, Indira Gandhi Medical College, Shimla, India; 8 Department of Cardiology, Christian Medical College, Ludhiana, India; 9 Department of Cardiology, Bombay Hospital and Medical Research Center, Mumbai, India; 10 Department of Cardiology, Sanjay Gandhi Post Graduate Institute of Medical Sciences, Lucknow, India; 11 Department of Cardiology, West Fort Hi-tech Hospital, Ltd., Trissur, India; 12 Department of Cardiology, Trinity Hospital and Heart Foundation, Bangalore, India; 13 Department of Cardiology, Sree Chitra Tirunal Institute for Medical Sciences and Technology, Trivandrum, India; 14 Department of Cardiology, King George Medical University, Lucknow, India; 15 Department of Cardiology, Christian Medical College, Vellore, India; University Heart Center, Germany

## Abstract

**Background:**

Studies from high-income countries have shown that women receive less aggressive diagnostics and treatment than men in acute coronary syndromes (ACS), though their short-term mortality does not appear to differ from men. Data on gender differences in ACS presentation, management, and outcomes are sparse in India.

**Methods and Results:**

The Detection and Management of Coronary Heart Disease (DEMAT) Registry collected data from 1,565 suspected ACS patients (334 women; 1,231 men) from ten tertiary care centers throughout India between 2007–2008. We evaluated gender differences in presentation, in-hospital and discharge management, and 30-day death and major adverse cardiovascular event (MACE; death, re-hospitalization, and cardiac arrest) rates. Women were less likely to present with STEMI than men (38% vs. 55%, p<0.001). Overall inpatient diagnostics and treatment patterns were similar between men and women after adjustment for potential confounders. Optimal discharge management with aspirin, clopidogrel, beta-blockers, and statin therapy was lower for women than men, (58% vs. 65%, p = 0.03), but these differences were attenuated after adjustment (OR = 0.86 (0.62, 1.19)). Neither the outcome of 30-day mortality (OR = 1.40 (0.62, 3.16)) nor MACE (OR = 1.00 (0.67, 1.48)) differed significantly between men and women after adjustment.

**Conclusions:**

ACS in-hospital management, discharge management, and 30-day outcomes did not significantly differ between genders in the DEMAT registry, though consistently higher treatment rates and lower event rates in men compared to women were seen. These findings underscore the importance of further investigation of gender differences in cardiovascular care in India.

## Introduction

India experienced more than 2.3 million deaths in 2008 due to cardiovascular diseases (CVD), and more than half of these deaths (1.3 million) were due to ischemic heart disease [1]. The CREATE registry of over 20,000 ACS patients across 89 centers in India showed that 30-day mortality for patients with ST-elevation myocardial infarction (STEMI) and for those with non-STEMI (NSTEMI) or unstable angina (UA) are 8.6% and 3.7%, respectively [2]. Previous ACS registries in India such as this have greatly informed our understanding of the presentation, management, and outcomes of individuals in India with ACS, but have not evaluated differences in gender.

Differences in gender are important to investigate because studies from high-income countries have repeatedly shown that women present differently and receive less intensive diagnostic and therapeutic management than men for ACS [3]–[10]. Specifically, women in the US with unstable angina are generally older, more likely to have a history of hypertension, and less likely to present with typical anginal symptoms than men [8]. Women with unstable angina are also less likely to receive recommended pharmacologic care or receive cardiac catheterization, coronary angioplasty, or bypass surgery compared to men [9]. Women in the US with non-ST elevation ACS have been shown to be less likely to receive acute heparin, angiotensin-converting enzyme inhibitors, and glycoprotein IIb/IIIa inhibitors than men, and less likely to receive aspirin, angiotensin-converting enzyme inhibitors, and statins at discharge [3]. Unfortunately, differences in the rate of reperfusion therapy and coronary angiography between men and women have not narrowed between 1994 and 2002 in the US [11]. Despite these differences in process of care measures, most studies have not shown any significant difference in outcomes between men and women, especially after adjustment for potential confounders [3], [5], [9], [10]. Similar patterns have been shown in Europe [12] and other regions of the world [13].

Given India’s history of gender relations and the gender differences that exist in the management of other diseases [14], it is reasonable to believe that gender differences in ACS management will also be present in India. We aimed to evaluate if such differences exist using data from the Detection and Management of Coronary Heart Disease (DEMAT) Registry, a prospective ACS registry with data from 1,565 ACS patients.

## Methods

### Study Population

The DEMAT study population included ACS patients from ten tertiary care centers across nine cities_throughout India. Six of these hospitals are private and four are public; six of the participating hospitals are academic teaching hospitals. Hospitals were selected based on willingness to participate in the registry. Consecutive patients who were admitted with suspected ACS between 2007 and 2008 underwent initial screening. Inclusion criteria consisted of: >18 years old and physician-diagnosed ACS, which was made based on the presence of chest pain consistent with ACS within the last 14 days and at least one of the following: ECG changes consistent with ACS, cardiac enzyme elevation, or a documented history of coronary heart disease [15]. Exclusion criteria included failure to provide informed consent, death from non-cardiac illness during hospitalization or participation in another clinical trial in which patient was receiving an investigational drug or device.

Medical history, anthropometric, clinical, and laboratory data were collected during hospitalization, upon discharge, and at 30 day follow-up, which occurred via in-person and telephonic interviews. Proxy respondents were used when participants were unavailable or had died.

### Informed Consent

Written informed consent was obtained from all study participants prior to collection of baseline information. Individual hospital ethics committees each approved the study protocol, namely: All India Institute of Medical Sciences (New Delhi, coordinating institution), Trinity Hospital and Heart Foundation (Bangalore), Christian Medical College (Ludhiana), Christian Medical College (Vellore), Monilek Hospital and Research Center (Jaipur), Bombay Hospital and Medical Research Center (Mumbai), West Fort Hi-tech Hospital Ltd (Trissur), Indira Gandhi Medical College (Shimla), King George Medical University (Lucknow), Sanjay Gandhi Post Graduate Institute of Medical Sciences (Lucknow), and Sree Chitra Tirunal Institute of Medical Sciences and Technology (Trivandrum).

### Statistical Analysis

Differences between men and women were compared using the *t* test for continuous variables and the χ^2^ test for categorical variables. Statistical significance was set at a p value <0.05. To assess the relationships between gender and in-hospital and discharge management, bivariate and multivariate logistic regression analyses were performed. The multivariate model included adjustment for age, institution, education, history of smoking, diabetes, hypertension, hyperlipidemia; history of coronary heart disease, stroke, or heart failure; history of aspirin, clopidogrel, beta-blocker, statin, ACE inhibitor or ARB; presenting ST segment elevation myocardial infarction; and prior angiography. Variables were identified by the bivariate analysis and then chosen based on clinical significance/plausibility. Bivariate and multivariate logistic regression analyses were also performed to assess the relationships between gender and risk for 30-day death and the combined endpoint of death, rehospitalization, and cardiac arrest at 30 days. The multivariate model for 30-day death included adjustment for age, education, history of CHD, current STEMI, or reperfusion of any type on current admission. The multivariate model for the 30-day composite endpoint included adjustment for age, education, smoking, history of diabetes, history of CHD, admission systolic blood pressure, admission heart rate, STEMI presentation, admission aspirin, admission clopidogrel, and reperfusion. All statistical analyses were performed using STATA 11.2 (StataCorp LP, College Station, TX).

## Results

### Baseline Characteristics

**Figure 1:**
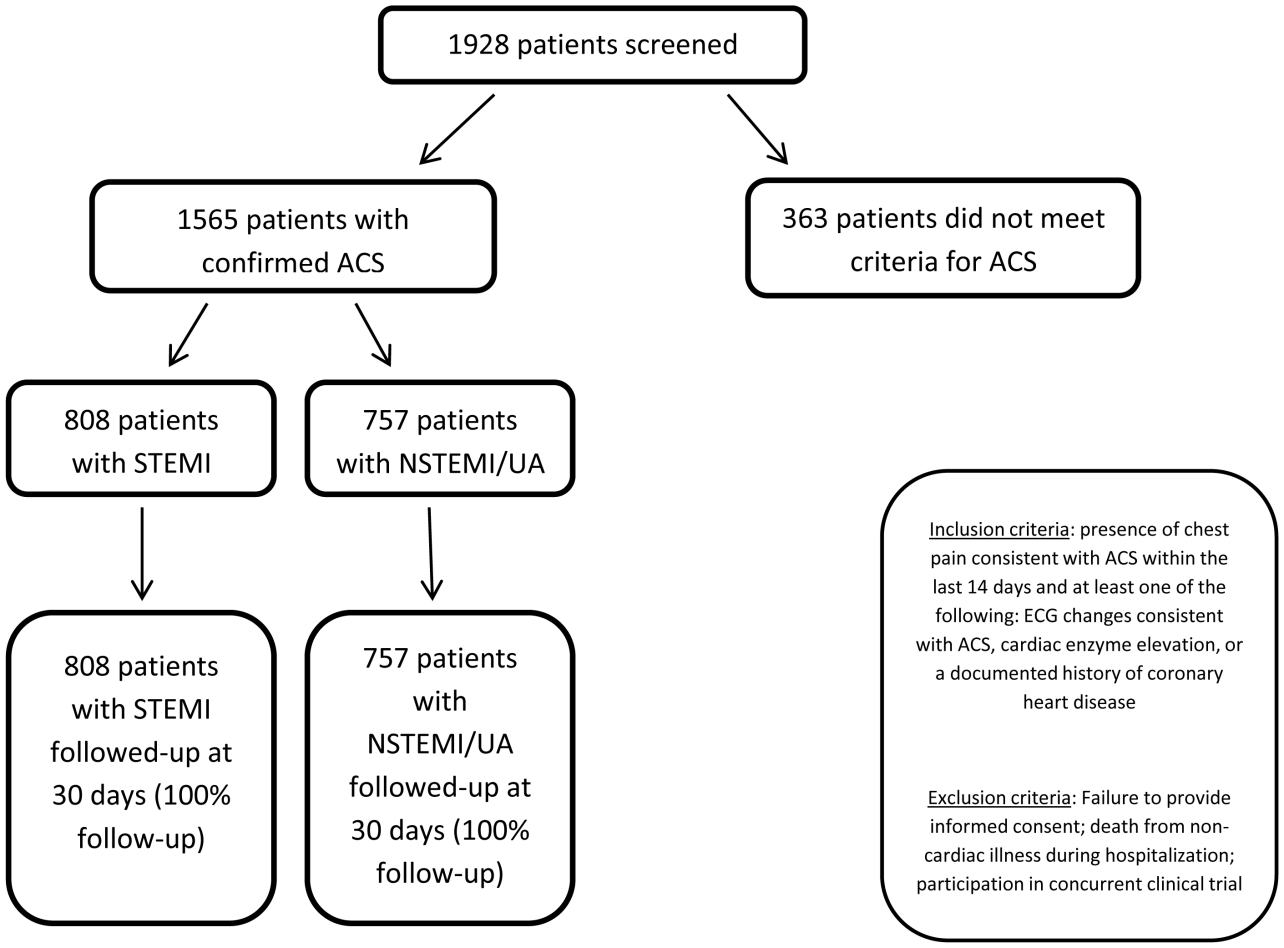
Flowchart of participants in DEMAT study.

Between 2007 and 2008, 1,928 consecutive patients were screened for suspected ACS at the ten participating centers; of those screened, 1,565 patients met eligibility criteria (Figure 1). Thirty day follow-up data were available for all participants.

More than half of patients presented with ST segment elevation myocardial infarction (STEMI, 52%). Approximately one in five (21%) ACS patients were women, whose mean age (60.8 years) was older than men (57.2 years; p<0.001). Women were also more likely than men to have a history of hypertension (62% vs. 42%; p<0.001) and diabetes (46% vs. 38%; p = 0.01) but were less likely to be tobacco users (2% vs. 33%; p<0.001). There were no gender differences in terms of history of hyperlipidemia, coronary heart disease (CHD), or stroke (Table 1). Women were as likely as men to present with prior aspirin (34% vs. 37%), clopidogrel (28% vs. 29%), beta-blocker (29% vs. 27%), ACE-inhibitor or ARB (24% vs. 19%), or statin (29% vs. 29%) use (p>0.05 for all). Women had a higher presenting systolic blood pressure (138.5 mmHg) compared with men (130.8 mmHg; p<0.001) but did not present with a higher diastolic blood pressure (82.7 vs. 81.6 mmHg; p = 0.14).

**Table 1:** Differences in demography/reported history, baseline medical therapy, and clinical presentation in patients with suspected ACS, stratified by gender among DEMAT participants.

	Men (n = 1231)	Women (n = 334)	p value
**Demography/Reported History**			
Age, years (SD)	57.2 (11.4)	60.8 (10.4)	<0.001
Education, years (SD)	12.8 (5.5)	9.8 (5.8)	<0.001
Body mass index, No. (kg/m^2^) (SD)	25.3 (4.8)	25.6 (4.2)	0.32
Hypertension, No. (%)	521 (42.3)	207 (62.0)	<0.001
Current tobacco, No. (%)	408 (33.1)	7 (2.1)	<0.001
Hyperlipidemia, No. (%)	185 (15.0)	52 (15.6)	0.81
Diabetes, No. (%)	467 (37.9)	152 (45.5)	0.01
Coronary heart disease, No. (%)	144 (11.7)	33 (9.9)	0.35
Stroke, No. (%)	19 (1.5)	3 (0.9)	0.37
**Baseline Medical Therapy**			
Aspirin, No. (%)	451 (36.6)	114 (34.1)	0.4
Clopidogrel, No.l (%)	359 (29.2)	95 (28.4)	0.8
Beta-blockers, No. (%)	331 (26.9)	97 (29.0)	0.43
ACE-I or ARB, No. (%)	234 (19.0)	79 (23.7)	0.06
Statin, No. (%)	351 (28.5)	98 (29.3)	0.77
**Clinical Presentation**			
STEMI, No. (%)	680 (55.2)	128 (38.3)	<0.001
Non-STEMI, No. (%)	551 (44.8)	206 (61.7)	<0.001
Systolic blood pressure, mmHg (SD)	130.8 (23.1)	138.5 (25.5)	<0.001
Diastolic blood pressure, mmHg (SD)	81.6 (12.0)	82.7 (13.2)	0.14
Heart Rate, beats per minute (SD)	81.4 (15.7)	81.2 (16.5)	0.78

### In-hospital Management

**Figure 2:**
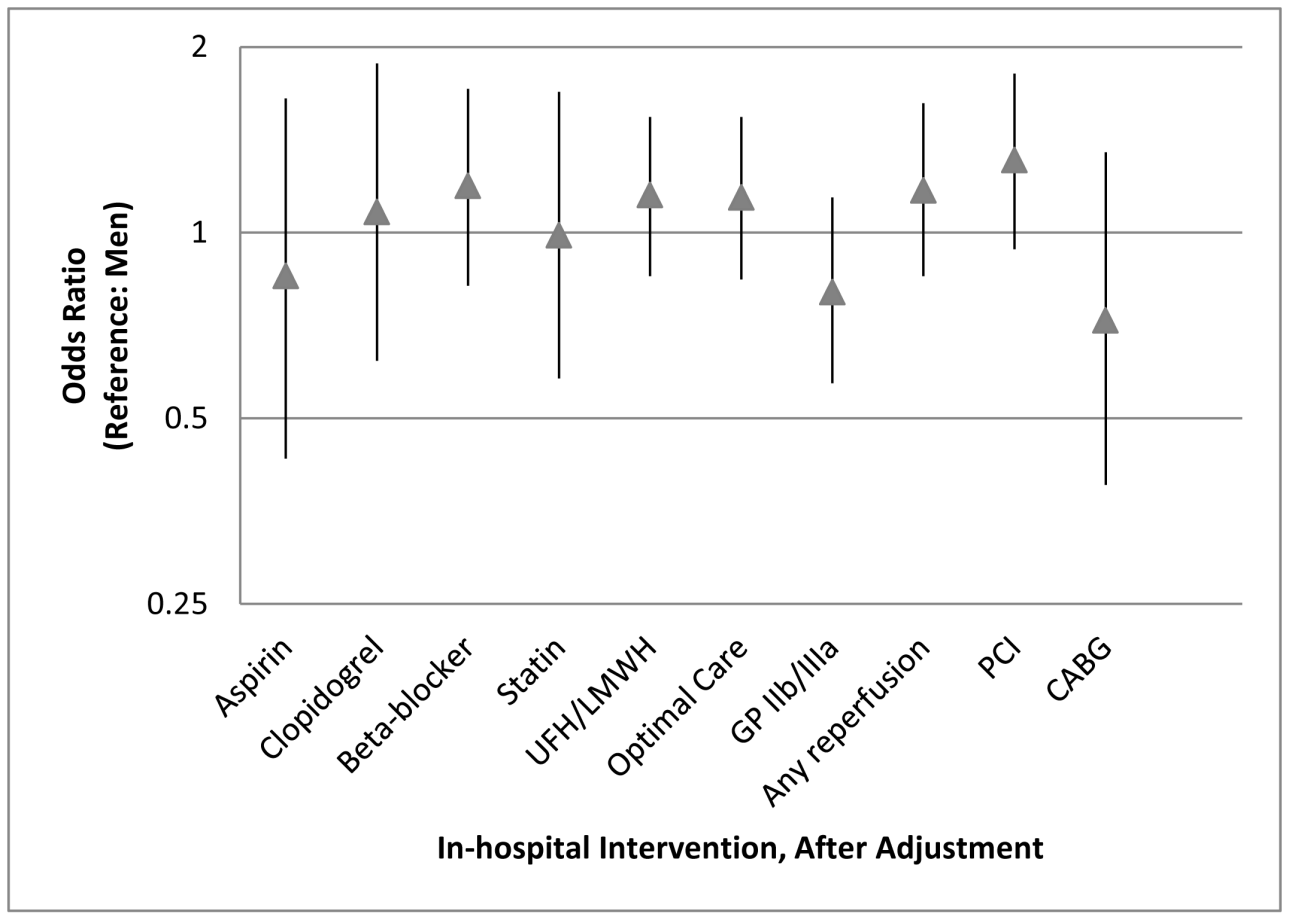
Association between gender and in-hospital management (OR 95% CI), after adjustment for age, institution, education, history of smoking, diabetes, hypertension, hyperlipidemia; history of coronary heart disease, stroke, or heart failure; history of aspirin, clopidogrel, beta-blocker, statin, ACE inhibitor or ARB; presenting ST segment elevation myocardial infarction; and prior angiography. Optimal care for in-hospital management includes administration of aspirin, clopidogrel, beta-blocker, statin, and unfractionated or low-molecular weight heparin. (UFH = unfractionated heparin; LMWH = low molecular weight heparin; GPIIb/IIIa = glycoprotein IIb/IIIa inhibitor; PCI = percutaneous coronary intervention; CABG = coronary artery bypass graft surgery).

Unadjusted in-hospital antiplatelet and statin use was high in both genders (>91% for aspirin, clopidogrel, and statin), but use of other guideline-based therapies was suboptimal (Table 2). Approximately three out of every four patients (79% for men vs. 76% for women; p = 0.42) received beta-blockers, and one out of every two patients (52% for men vs. 52% for women; p = 0.85) received unfractionated heparin or low molecular weight heparin. Two out of every three patients received some form of reperfusion therapy (68% in men vs. 64% for women; p = 0.11). Women were less likely to receive glycoprotein IIb/IIIa inhibitors (GP IIb/IIIa) (27% for men vs. 22% for women; p = 0.05) or to receive thrombolysis (25.3% for men vs. 16.2% for women; p<0.01) compared to men. Women were as likely to receive an optimal in-hospital ACS regimen of aspirin, clopidogrel, beta-blocker, statin, and either low molecular weight heparin or unfractionated heparin, though combined rates of all medicines for both genders were low (38% for men vs. 36% for women; p = 0.51). Figure 2 demonstrates gender differences in in-hospital management after adjusting for potential confounders. After adjustment, no differences were seen in in-hospital management between women and men.

**Table 2:** Differences in in-hospital management and discharge management, stratified by gender among DEMAT participants.

	Men (n = 1231)	Women (n = 334)	p value
**In-hospital Management**			
Aspirin, No. (%)	1183 (96.1)	313 (93.7)	0.06
Clopidogrel, No. (%)	1154 (93.7)	309 (92.5)	0.42
Beta-blocker, No. (%)	975 (79.2)	255 (76.4)	0.26
Statin, No. (%)	1150 (93.4)	306 (91.6)	0.25
UFH/LMWH, No. (%)	634 (51.5)	174 (52.1)	0.85
GPIIb/IIIa Inhibitor, No. (%)	340 (27.6)	74 (22.2)	0.05
Any reperfusion, No. (%)	841 (68.4)	213 (63.8)	0.11
Thrombolysis, No. (%)	310 (25.3)	54 (16.2)	<0.01
PCI, No. (%)	549 (49.0)	155 (49.4)	0.90
CABG, No. (%)	73 (6.6)	18 (5.8)	0.61
Optimal care, No. (%)*	463 (37.6)	119 (35.6)	0.51
**Discharge Management**			
Aspirin, No. (%)	1124 (93.9)	303 (94.4)	0.74
Clopidogrel, No. (%)	1098 (91.8)	280 (87.2)	0.01
Beta-blockers, No. (%)	958 (80.0)	248 (77.3)	0.28
Statins, No. (%)	1057 (88.5)	275 (86.2)	0.26
ACE-I or ARB, No. (%)	824 (66.9)	211 (63.2)	0.20
Optimal care, No. (%)**	774 (64.7)	187 (58.3)	0.03

* Optimal care for in-hospital management includes administration of aspirin, clopidogrel, beta-blocker, statin, and unfractionated or low-molecular weight heparin.

** Optimal care for discharge management includes administration of aspirin, clopidogrel, beta-blocker, and statin.

### Discharge Management

**Figure 3:**
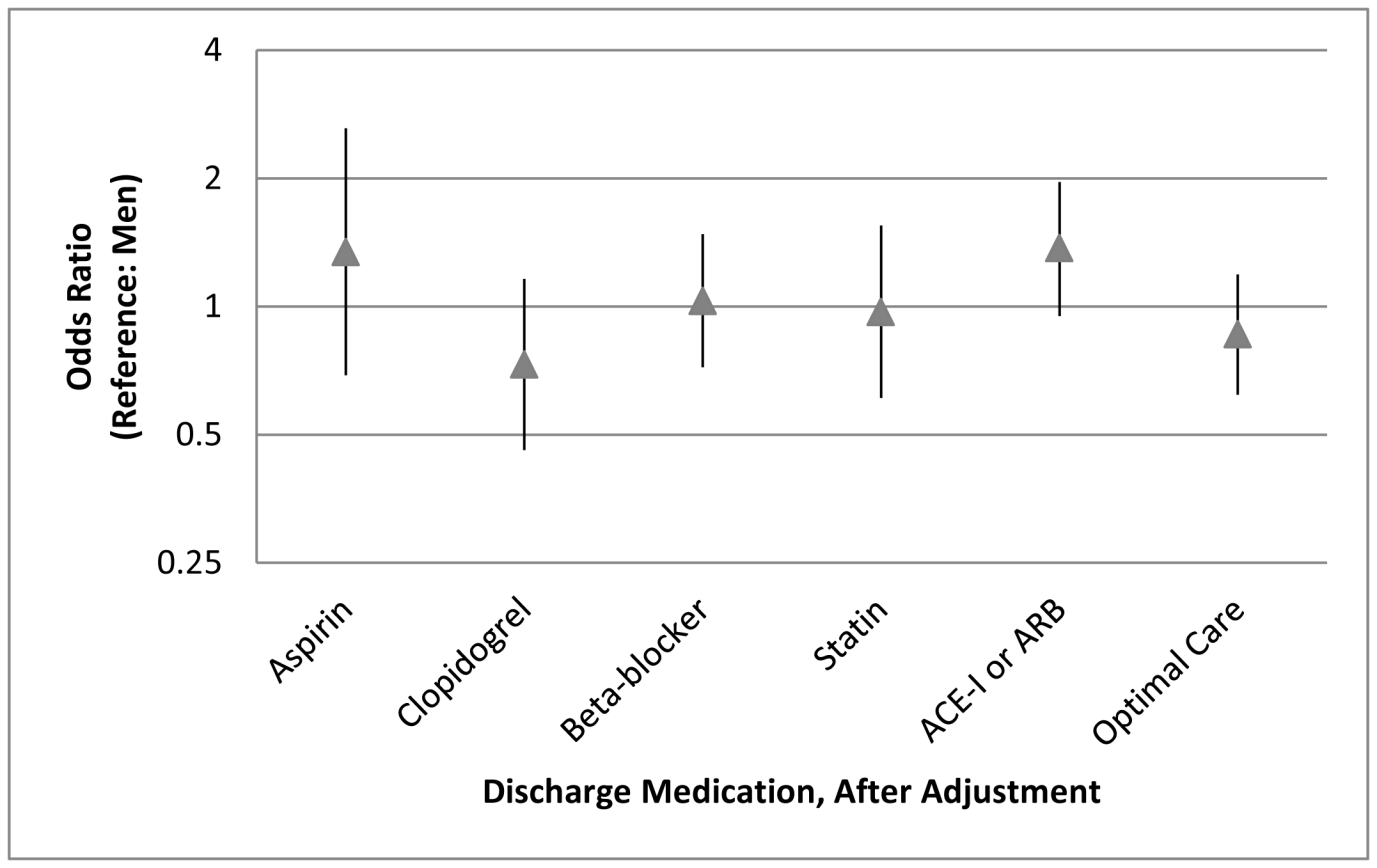
Association between gender and discharge medications (OR 95% CI), after adjustment for age, institution, education, history of smoking, diabetes, hypertension, hyperlipidemia; history of coronary heart disease, stroke, or heart failure; history of aspirin, clopidogrel, beta-blocker, statin, ACE inhibitor or ARB; presenting ST segment elevation myocardial infarction; and prior angiography. Optimal care for discharge management includes administration of aspirin, clopidogrel, beta-blocker, and statin. (UFH = unfractionated heparin; LMWH = low molecular weight heparin; GPIIb/IIIa = glycoprotein IIb/IIIa inhibitor; PCI = percutaneous coronary intervention; CABG = coronary artery bypass graft surgery; ARB = angiotensin receptor blocker).

Whereas unadjusted rates of aspirin prescription at discharge were similar between men and women (94% for men; 94% for women; p = 0.74), the unadjusted rates of clopidogrel prescription at discharge were higher in men compared with women (92% for men vs. 87% for women; p = 0.01). More than four out of every five discharged patients were prescribed statins (89% for men vs. 86% for women; p = 0.28) with similar rates of beta-blocker (80% for men vs. 77% for women; p = 0.28) and slightly lower rates of overall ACE-I or ARB prescriptions (67% for men vs. 63% for women; p = 0.20). At discharge, women were less likely than men to receive a combined, optimal care regimen for ACS secondary prevention that includes aspirin, clopidogrel, beta-blockers, and statin therapy (65% for men vs. 58% for women, p = 0.03). Figure 3 demonstrates gender differences in discharge medication prescription rates after adjusting for potential confounders. After adjustment, no differences were seen in discharge medication prescription between women and men.

### Outcomes

At 30-day follow-up, 10 women (3.0%) and 22 men (1.8%) died (unadjusted OR = 1.70 (0.80, 3.64), Table 3 and Table 4). After adjustment for potential confounders, this estimate was attenuated but remained similar (OR = 1.40 (0.62, 3.16), Table 4). For the combined endpoint of death, rehospitalization, or cardiac arrest at 30 days, 45 women (13.5%) and 154 men (12.5%) experienced one of these events (unadjusted OR = 1.09 (0.76, 1.56), Table 3 and Table 4). After adjustment for potential confounders, this association was attenuated but remained similar (OR = 1.00 (0.67, 1.48), Table 4). Our results remained similar after sensitivity analyses, which included stratifying the analysis of each outcome by institution and creating random effects regression models to adjust for within-institution clustering. No age-gender interaction was seen in the mortality outcome or the composite outcome using either 65 years-old or 75 years-old as the cut-off.

**Table 3:** 30-day outcomes among DEMAT participants, stratified by gender.

Outcome	Men (n = 1231)	Women (n = 334)	p value
Death at 30 days, No. (%)	22 (1.8)	10 (3.0)	0.16
Death, rehospitalization, or cardiac arrest at 30 days, No. (%)	154 (12.5)	45 (13.5)	0.64

**Table 4:** Association between gender and 30-day outcomes of DEMAT participants (reference: men).

Outcome		Model 1*		Model 2**		Model 3†	
**Death at 30 days (OR (95%CI))**		1.70 (0.80, 3.64)		1.44 (0.67, 3.10)		1.40 (0.62, 3.16)	
**Death, rehospitalization, or cardiac arrest at 30 days (OR (95%CI))**	1.09 (0.76, 1.56)		1.03 (0.72, 1.48)		1.00 (0.67, 1.48)		

* Model 1: Unadjusted logistic regression model.

** Model 2: Logistic regression model adjusted for age.

† Model 3: Multivariate logistic regression model for 30-day mortality includes adjustment for age, education, history of CHD, current STEMI, or reperfusion of any type on current admission. Model for 30-day composite endpoint includes adjustment for age, education, smoking, history of diabetes, systolic blood pressure on admission, heart rate on admission, history of CHD, current STEMI, reperfusion of any type on current admission, administration of aspirin on admission, or administration of clopidogrel on admission.

## Discussion

In this registry of 1,565 patients with suspected ACS from ten tertiary care centers, we present the first data regarding gender differences in presentation, management, and outcome of ACS in India. Our results show that on presentation, women were more likely to be older and to have a history of hypertension and diabetes than men, though they were less likely to be tobacco users. After adjustment for possible confounding factors, there were no significant differences between men and women in in-hospital and discharge management for ACS. There were also no significant differences in the outcome of death or in the composite outcome of death, rehospitalization, or cardiac arrest at 30 days after adjustment.

Regarding gender differences, there are limited data available from other ACS registries in low- and middle-income countries, including India. The OASIS 1 and 2 and CREATE ACS registries in India have not explored gender differences in presentation, management, and outcomes [2], [15]. A 2007 ACS registry of 1,301 patients from 12 medical teaching hospitals in China found lower rates of reperfusion and higher rates of recurrent angina in women, but in-hospital mortality rates were similar [16]. A 2011 Egyptian ACS registry of 1,204 patients from five hospitals revealed that women were less likely to receive aspirin on admission, anticoagulant therapy or angiography during hospitalization, or aspirin or statin therapy at discharge [17]. However, these differences in management were not associated with a difference in in-hospital mortality after adjustment for baseline characteristics, despite higher event rates. Finally, a 2007 Thai registry of 3,836 patients with STEMI from 17 hospitals found that beta-blockers, statins, ACEI therapy, ARB therapy, coronary angiography, thrombolysis, and PCI were used less frequently in women [13]. However, after adjustment for multiple covariates, there was no difference in in-hospital mortality between groups.

Several studies from high-income countries have also shown a difference in ACS management between men and women. In the American College of Cardiology – National Cardiovascular Data Registry study of 199,690 patients with ACS, women received aspirin and GP IIb/IIIa inhibitors and were discharged on aspirin and statin therapy less often than men [18]. Even though women had fewer high-risk features on angiography, they were more likely to incur in-hospital complications than men. However, the rate of in-hospital mortality was similar between genders. The American Heart Association’s Get With the Guidelines Coronary Artery Disease database of 78,254 patients from the US showed that women were less likely to receive early aspirin and beta-blocker therapy, reperfusion therapy, timely reperfusion, cardiac catheterization, and revascularization procedures after an acute MI [19]. Among patients with STEMI alone, women had a higher in-hospital mortality rate. The Global Registry of Acute Coronary Events examined 26,755 patients in 14 mostly high-income countries in Europe, the Americas, and the South Pacific [20]. This registry revealed that women with advanced disease were treated less aggressively than their male counterparts, and that women were more likely to have adverse outcomes (death, MI, stroke, rehospitalization) than men. However, no difference was seen in mortality alone after adjustment.

In contrast to the above studies both in LMICs and in high-income countries, our registry demonstrated no significant difference in in-hospital management of ACS. On the other hand, our data are consistent with many of the studies mentioned above which do not show a difference in mortality between genders, despite differences in process-of-care measures. The lack of mortality difference in these studies could potentially be due to an age-gender interaction, which has been seen in several prior U.S. studies [21]–[23]. In these studies, women below a certain age (either 65 years or 75 years old) had a significantly higher mortality due to ACS compared to men, but older women did not. In our study, we found no such age-gender interaction, at either the 65 year-old or 75 year-old threshold (data not shown). However, our event rates may have been too low to detect a meaningful age-gender interaction.

Currently, there is a dearth of information about gender differences with regard to management and outcomes of various diseases in India, particularly CVD. One prescription audit-based study of 1,602 prescriptions in Rajasthan, India examined disparities in treatments for secondary prevention of coronary artery disease. The authors found that with the exception of a modestly decreased use of beta-blockers in women, there was no significant difference between genders [24]. Though this is consistent with the results from our registry, it should be noted that this prescription-based study had a small sample size and is limited to individuals who had access to pharmacies and medical care, which may differ between men and women. While studies have been published with regard to gender differences in HIV management [25], little attention has been paid thus far to gender disparities for chronic diseases such as CVD, which will become increasingly relevant as CVD prevalence increases in India. Even an issue as fundamental as the ratio of men to women who present to the hospital with ACS, which appears to be decreasing over time in India [26]–[28], raises questions as to whether this is due to a change in incidence pattern of ACS in men and women versus a change in access to care between genders, underscoring how little is known about gender differences in ACS in India.

Our study has several limitations. First, our sample size was relatively modest, and there were relatively few events to compare differences in outcomes. Thus, we may not have had sufficient power to detect important gender differences in short-term outcomes that may be present throughout all ACS patients in India. In addition, our follow-up time of 30 days may not have been long enough to detect a true divergence in outcomes. Nonetheless, this study is the first of its kind in India and represents a first step in understanding and addressing potential gender differences in ACS in India. Second, our study included patients from a limited number of urban, tertiary care centers and may not be generalizable to other centers in India, particularly those outside of the urban setting. Third, our data are observational, which are susceptible to residual confounding, despite our attempts to control for potential confounders in our regression models. Fourth, we did not collect data on relative or absolute contraindications for medical therapy, which may lead to an underestimate of appropriate in-hospital and discharge treatment rates.

In conclusion, this study of 1,565 patients with suspected ACS from ten tertiary care centers in India is, to the best of our knowledge, the first of its kind to examine gender differences in ACS presentation, management, and outcomes in India. No significant differences in in-hospital management, discharge management, or 30-day outcomes were seen between genders after adjustment for potential confounders. As India stands on the precipice of a cardiovascular disease epidemic, further investigations with larger sample sizes in varied clinical settings are urgently needed to shed light on possible differences in the management and outcomes of women vs. men with ACS.
